# Expenses of hospitalization for ambulatory care sensitive conditions in the Health Regional Offices of the State of São Paulo

**DOI:** 10.31744/einstein_journal/2021GS5817

**Published:** 2021-07-23

**Authors:** Bruna Moreno Dias, Ariane Cristina Barboza Zanetti, Antonio Carlos Pereira

**Affiliations:** 1 Universidade de São Paulo Escola de Enfermagem de Ribeirão Preto Ribeirão PretoSP Brazil Escola de Enfermagem de Ribeirão Preto, Universidade de São Paulo, Ribeirão Preto, SP, Brazil.; 2 Universidade Estadual de Campinas Faculdade de Odontologia de Piracicaba PiracicabaSP Brazil Faculdade de Odontologia de Piracicaba, Universidade Estadual de Campinas, Piracicaba, SP, Brazil.

**Keywords:** Primary Health Care, Hospitalization, Hospital costs, Health expenditures, Regional health planning

## Abstract

**Objective:**

To analyze the expenses of hospitalizations for ambulatory care sensitive conditions in the Health Regional Offices of the State of São Paulo.

**Methods:**

An ecological, retrospective cohort, with analysis of hospital admissions for ambulatory care sensitive conditions in the state of São Paulo, from 2014 to 2018, compiled by the Hospital Information System. Data were extracted using the Tabwin application and analyzed using descriptive statistics.

**Results:**

There was a 14.49% reduction in the amount spent on hospitalizations for ambulatory care sensitive conditions. There were reductions in the frequency of hospitalizations (−1.26) and hospital stay (−0.54), and an increase in the occurrence of deaths (8.02). The Regional Offices of Barretos, Taubaté and Araraquara showed an increase in expenses in the period, by 37.86%, 15.38% and 3.78%, respectively, while all other Regional Offices showed a reduction; in that, the most significant were in Bauru (−31.90%), São João da Boa Vista (−26.18%), Presidente Prudente (−21.00%) and São Paulo (−19.17%). The value of hospitalizations for ambulatory care sensitive conditions showed a strong and positive correlation with the variables frequency and hospital stay.

**Conclusion:**

The results pointed to a difference in the amounts spent on hospitalizations for ambulatory care sensitive conditions in the Regional Offices, although there was no difference in the frequency and duration of these hospitalizations. The expansion of Primary Health Care resources is a possible element for reducing the frequency and spending on hospitalizations for ambulatory care sensitive conditions; nonetheless, it is necessary to consider other factors, such as social determinants and the organization of health services.

## INTRODUCTION

Primary Health Care (PHC) is understood as the main strategy to expand access and change the care model of the Brazilian Unified Health System (SUS - *Sistema Único de Saúde*). It is also structured to be the guiding axis of this system.^(^^1^^)^

Among the attributes of PHC is the resolution capacity, linked to measures of health promotion, disease prevention and treatment, so that early detection of diseases and their proper treatment do not progress to services at other levels of care, reducing the number of hospitalizations. This foundation supports the proposition of the indicator of hospitalizations for ambulatory care sensitive conditions (ACSC).^(^^2^^)^

The hospitalizations for ACSC can be used as part of the evaluation of the resolvability, quality, and access to PHC, helping to identify the need, reorientation, and proposition of public health policies. In a supplementary way, the indicator may reflect the inappropriate use of health services,^(^^3^^)^ or even show changes in the model of care and in the pattern of health financing.^(^^4^^)^

The indicator has been used in several countries. In Brazil, a national list of hospitalizations for ACSC was proposed in 2008. It is structured into groups of causes for hospitalizations and diagnoses, with the objective of being an instrument to evaluate PHC and/or the use of hospital care, as well as performance of the health system in the different levels of management.^(^^5^^)^

Assessing the health system should occur in parallel with the discussion and understanding of health financing, which has been insufficient to ensure a universal, comprehensive, and quality system. Given this need, understanding the performance of health services and the relation with health expenditures is an important possibility to optimize the system. Moreover, in the context of hospitalizations for ACSC, the reduced spending on hospital admissions allows reinvestment in the system.^(^^4^^)^

The Brazilian list of sensitive conditions includes highly relevant diagnoses in health care of the population. Thus, by analyzing the reality of each region, the monitoring of profile, and the strategies of control and reduction of hospitalizations for ACSC make it possible to adopt measures to restructure the organization and operation of hospital services, facing demands of high complexity of care.

Thus, the analysis of resources used in hospitalizations for ACSC aims to provide subsidies for planning, management, and evaluation of health services, programs, and policies - especially for situations of greater vulnerability and inequity.

## OBJECTIVE

To analyze the expenses with hospitalizations for ambulatory care sensitive conditions in the Health Regional Offices of the State of São Paulo.

## METHODS

This is an ecological, retrospective cohort study, having as outcome the occurrence of hospital admissions for users with a primary diagnosis on the Brazilian list of ACSC, in the Health Region Offices of the State of São Paulo.

We considered eligible all hospitalizations that occurred in the state of São Paulo during the period from 2014 to 2018, compiled by the Hospital Information System (SIH - *Sistema de Informações Hospitalares*) of SUS, with data structured in the 17 Health Regional Offices of the state. They are Greater São Paulo, Araçatuba, Araraquara, Baixada Santista, Barretos, Bauru, Campinas, Franca, Marília, Piracicaba, Presidente Prudente, Registro, Ribeirão Preto, São João da Boa Vista, São José do Rio Preto, Sorocaba, and Taubaté.

The five-year period was considered, with 2018 as the most recent consolidated data available for public consultation at the time of the search. The year 2014 was considered as a reference for value adjustment, according to the inflation of the period.

We used secondary source data from the microdata of the SIH from the SUS Information Technology Department (DATASUS - *Departamento de Informática do* SUS), through public consultation in the form of reduced files, and the Tabwin application version 4.1.5, developed and made available by DATASUS.

The identification of hospitalizations for sensitive conditions was based on the list of diagnoses according to the International Classification of Diseases and Health-Related Problems (ICD-10), contained in the Brazilian list of hospitalizations for ACSC, provided by the Administrative Ruling 221 of the Ministry of Health, of April 17, 2008.^(^^5^^)^

The records obtained were exported to Microsoft Excel software, composing a database on an electronic spreadsheet. For statistical analysis, the IBM SPSS Statistics software, version 19, was used. Linear correlation was employed to analyze the relation between numerical variables; and for comparison between 2014 and 2018, Wilcoxon's paired test was used, with a significance level of 5%.

## RESULTS

In the period under study, the increase in overall hospitalizations and in the population of the state were observed, together with a drop in hospitalizations for ACSC (Table 1).

**Table 1:** Proportion and rate of hospitalizations for ambulatory care sensitive conditions in the State of São Paulo

Year	Hospitalizations for ACSC	General hospital admissions	Proportion of hospitalizations for ACSC (%)	Population	Hospitalizations for ACSC rate (per thousand)
2014	377,988	2,496,141	15.14	43,937,755	8.60
2015	377,752	2,493,368	15.15	44,356,304	8.52
2016	372,974	2,489,614	14.98	44,760,305	8.33
2017	373,069	2,498,888	14.93	45,149,603	8.26
2018	373,241	2,538,337	14.70	45,538,936	8.20

ACSC: ambulatory care sensitive conditions.

**Figure 1:**
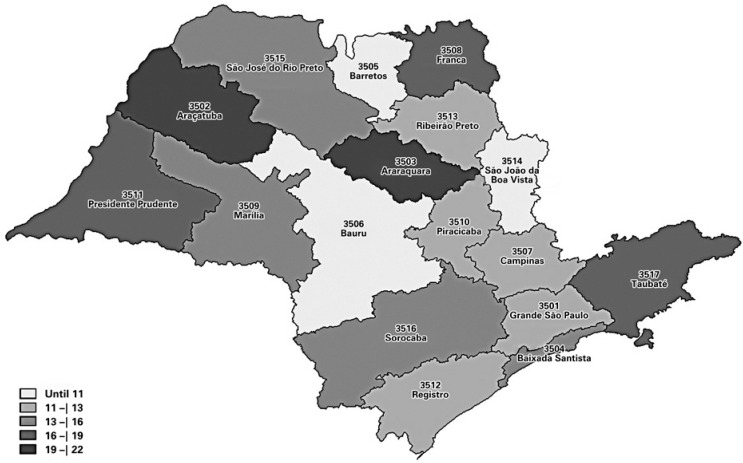
Proportion of spending on hospitalizations for ambulatory care sensitive conditions in relation to expenses on general hospital admissions in 2018

The amounts spent in hospitalizations for ACSC accounted for 12.72% of value of general hospital admissions. As shown in figure 1, the Regional Offices with the lowest expenditure proportions in relation to general hospitalizations were Barretos, Bauru, and São João da Boa Vista; the ones with the highest proportions were Araraquara, Araçatuba, and Presidente Prudente.

In absolute terms, the frequency of hospitalizations for ACSC decreased in 11 of the 17 Regional Offices, with Bauru, São João da Boa Vista, and Piracicaba showing the greatest reduction. The increase in frequency occurred mainly in the Taubaté and Araraquara Regional Offices, as shown in table 2. When comparing 2014 and 2018, no significant difference was observed in the frequency of hospitalizations for ACSC (p=0.435).

**Table 2:** Frequency, length of stay, deaths and amounts spent on hospitalizations for ambulatory care sensitive conditions per Health Regional Office in the State of São Paulo

Heath regional offices	Frequency				Value (R$)				Length of hospital stay				Death			
	2014	2018	Variation(%)	p value*	2014†	2018	Variation(%)	p value*	2014	2018	Variation(%)	p value*	2014	2018	Variation(%)	p value*
Greater São Paulo	151,404	154,241	1.87	0.717	225,575,520.73	182,330,045.28	−19.17	0.005‡	1,107,765	1,050,257	−5.19	0.398	9,167	9,991	8.99	0.396
Araçatuba	9,179	8,505	−7.34	0.794	8,696,477.00	8,522,201.50	−2.00	0.198	39,109	41,163	5.25	0.421	584	625	7.02	0.849
Araraquara	10,418	11,489	10.28	0.121	14,189,798.98	14,726,521.44	3.78	0.277	76,060	69,921	−8.07	0.184	746	778	4.29	0.192
Baixada Santista	10,470	9,914	−5.31	0.494	12,191,048.68	12,117,636.75	−0.60	0.968	73,034	75,188	2.95	0.778	930	1,109	19.25	0.067
Barretos	5,425	5,197	−4.20	0.184	3,688,118.69	5,084,465.88	37.86	0.024‡	21,402	26,623	24.39	0.103	272	362	33.09	0.199
Bauru	23,963	19,894	−16.98	0.014‡	30,225,019.28	20,583,674.20	−31.90	0.005‡	132,591	120,284	−9.28	0.147	1,488	1,306	−12.23	0.133
Campinas	29,171	30,844	5.74	0.117	39,535,158.53	34,331,818.73	−13.16	0.126	174,020	190,628	9.54	0.036‡	1,806	1,855	2.71	0.672
Franca	7,024	6,965	−0.84	0.717	10,141,339.43	8,417,055.31	−17.00	0.243	30,425	30,921	1.63	0.872	477	387	−18.87	0.033‡
Marília	16,616	15,180	−8.64	0.334	17,601,805.86	15,780,963.18	−10.34	0.030‡	83,783	88,190	5.26	0.376	1,026	1,001	−2.44	0.460
Piracicaba	10,146	9,010	−11.20	0.067	16,382,931.56	13,300,291.47	−18.82	0.126	56,661	52,696	−7.00	0.376	720	731	1.53	0.536
Presidente Prudente	13,314	13,512	1.49	0.841	14,014,865.71	11,071,972.62	−21.00	0.099	60,237	61,590	2.25	0.546	733	712	−2.86	0.950
Registro	1,911	1,810	−5.29	0.931	1,493,468.36	1,376,145.93	−7.86	0.687	10,682	11,551	8.14	0.171	273	354	29.67	0.043‡
Ribeirão Preto	15,184	15,744	3.69	0.421	26,336,769.31	22,298,947.30	−15.33	0.005‡	88,465	94,897	7.27	0.243	834	842	0.96	0.652
São João da Boa Vista	10,557	9,138	−13.44	0.023‡	8,568,923.81	6,325,339.92	−26.18	<0.001‡	47,913	45,956	−4.08	0.520	850	719	−15.41	0.170
São José do Rio Preto	26,039	23,477	−9.84	0.212	37,273,150.84	34,250,673.03	−8.11	0.968	109,751	115,225	4.99	0.629	924	1,440	55.84	0.024‡
Sorocaba	18,265	17,112	−6.31	0.573	20,268,370.15	17,507,823.74	−13.62	0.070	85,730	93,699	9.30	0.398	1,657	1,770	6.82	0.338
Taubaté	18,902	21,209	12.21	0.243	25,885,826.35	29,866,293.97	15.38	0.968	146,653	162,927	11.10	0.084	1,434	1,858	29.57	0.016‡
Total	377,988	373,241	−1.26	0.435	512,068,592.46	437,891,870.25	−14.49	0.010‡	2,344,281	2,331,716	−0.54	0.266	23,921	25,840	8.02	0.107

* Wilcoxon paired test, statistical significance considered for p<0.05

† 2014 value adjusted for inflation in the period

‡ p<0.05.

For the amounts spent, a significant difference was observed between the years under study (p=0.010), with a reduction by −14.49%, when comparing the amount spent in 2018, with that in 2014, adjusted for inflation. Only the Regional Offices of Barretos, Taubaté, and Araraquara had increased spending during the period, by 37.86%, 15.38%, and 3.78%, respectively. The most significant reductions were in Bauru (−31.90%), São João da Boa Vista (−26.18%), Presidente Prudente (−21.00%), and São Paulo (−19.17%). A significant difference was observed for the amounts spent in the regions of Greater São Paulo (p=0.005), Barretos (p=0.024), Bauru (p=0.005), Marília (p=0.030), Ribeirão Preto (p=0.005), and São João da Boa Vista (p<0.001).

Although there was a slight reduction in hospital stay (−0.54%), some Regional Offices showed an increase of more than 10%, such as Barretos (24.39%) and Taubaté (11.10%). The Regional Offices with the greatest reductions in length of stay were Bauru (−9.28%), Araraquara (−8.07%), and Piracicaba (−7.00%).

An increase in the occurrence of deaths was observed in the state, and the Regional Offices with the most expressive increases were São José do Rio Preto (55.84%), Barretos (33.09%), Registro (29.67%), and Taubaté (29.57%). On the other hand, the reductions were in Franca (−18.87%) and São João da Boa Vista (−15.41%).

No significant differences were observed between 2014 and 2018 in the length of hospital stay (p=0.266) and in the occurrence of deaths (p=0.107).

The Regional Offices Bauru and São João da Boa Vista showed uniform behavior in reduction of frequency, value, hospital stay, and deaths. Taubaté experienced an increase in all variables and Barretos, although with a reduction in the frequency of hospitalizations, had an increase in other variables, as the Regional Office with the most expressive increases in the value spent and length of hospital stay.

In addition, using 2018 as a reference, a strong and positive correlation was identified between the frequency and the value of hospitalizations for ACSC (r=0.997), between hospital stay and the value of hospitalizations for ACSC (r=0.996), and between the frequency of hospitalizations and the occurrence of deaths from ambulatory care sensitive conditions (r=0.994).

## DISCUSSION

In the period under study, we observed a reduction in the proportion (from 15.14 to 14.70) and in rate of hospitalizations for ACSC (from 8.60 to 8.20) simultaneously with the expansion of the installed PHC capacity, demonstrated by the increased number of Family Health Strategy (FHS) teams from 3,380 in 2010, to 5,257 in 2018. Additionally, there was greater coverage during the same period by FHS and Primary Care teams - from 28.29% to 41.23%, and from 82.55 to 85.96%, respectively.^(^^6^^)^

The observed rates are similar to those of cities with high primary care coverage, such as Florianópolis and Curitiba, with values close to 10%, while the Federal District presented a value close to 13%.^(^^7^^)^

The proportion of hospitalizations for ACSC in relation to general hospitalizations is also lower than that of other studies, as observed in Goiás, where 30% of admissions were for ambulatory care sensitive conditions,^(^^8^^)^ 25.3% in the state of Mato Grosso do Sul,^(^^9^^)^ and 32.4% in the city of Itaboraí (RJ).^(^^10^^)^

In 2018, hospitalizations for ACSC accounted for 12.72% of total spending on admissions. This behavior was also observed in São Leopoldo (RS), which, in 2012, allocated 15.80% of spending on hospitalizations for ACSC.^(^^11^^)^

The increase in values at the Barretos, Taubaté, and Araraquara Regional Offices is noteworthy. In the Regional Office of Barretos, there is an increase in the amount spent, length of stay, and deaths due to ACSC, although there has been a reduction in the frequency of hospitalizations. The Regional Office has 2.05 beds for one thousand inhabitants, that is, a higher proportion among the Regional Offices. It has a coverage of 58.81% of FHS teams and a coverage of 107.24% of PHC.^(^^6^^)^ Despite the increased amount spent on hospitalizations for ACSC, in 2018, the Regional Office committed 7.89% of amount spent on general hospitalizations, which is the lowest proportion observed among the Regional Offices.

In the Regional Office of Taubaté, there was an increase in the amount spent, accompanied by an increase in frequency, length of stay, and deaths due to ACSC. The region has 1.29 beds per one thousand inhabitants, with 48.37% of FHS coverage and 96.38% of PHC coverage,^(^^6^^)^ using 16.13% of values spent on hospitalizations for ACSC.

Similarly, the Regional Office of Araraquara showed a reduction in length of hospital stay for ACSC, although it had high frequency, value, and deaths. The Regional Office has 1.38 beds per one thousand inhabitants, FHS coverage of 45.84%, and PHC coverage of 99.65%.^(^^6^^)^ It is the Regional Office with the highest expenditure, when compared to the values of general hospitalizations, with 21.51%.

Among the Regional Offices where there was reduction in the amounts spent, Bauru and São João da Boa Vista stood out. In both, the reduction in the amounts spent follows the reduction in frequency, length of stay, and deaths due to ACSC.

In São João da Boa Vista, there are 1.48 beds per thousand inhabitants, and the FHS coverage is 46.4%, whereas the PHC coverage is 92.65%.^(^^6^^)^ The hospitalizations for ACSC among general hospitalizations accounted for 10.33%. In Bauru, there are 1.88 beds per thousand inhabitants, 43.35% of FHS coverage, and 102.45% of PHC coverage.^(^^6^^)^ The hospitalizations for ACSC among general hospitalizations accounted for 9.87%.

In a previous study carried out in the Regional Offices of the state of São Paulo, from 2000 to 2007, there was a reduction in the frequency of hospitalizations for ACSC in almost all Regional Offices, except in the Greater São Paulo, Araçatuba, and Ribeirão Preto.^(^^12^^)^ In this study, São Paulo and Ribeirão Preto kept an upward trend in the frequency of hospitalizations for ACSC, whereas Araçatuba showed a reduction. For the period from 2000 to 2007, the largest reductions were reported in the Regional Offices of Barretos, Araraquara, and Taubaté,^(^^12^^)^ which in this study, with the exception of Barretos, experienced an increased frequency.

In terms of structure, all the Regional Offices showed an expansion of PHC resources, with an increase in FHS coverage from 28.29%, in 2010, to 41.23%, in 2018. There were also important differences among the Regions, as can be seen in the minimum and maximum numbers for 2018 in the Regional Offices under study, for FHS (72 to 2,062), coverage of FHS (30.46% to 108.29%), number of physicians in PHC (130 to 4,995), and coverage of PHC teams (71.85% to 140.70%).^(^^6^^)^

This behavior follows the expansion of PHC teams in the country over the last 20 years, in which the FHS coverage has increased from 4.4% to 70%, with a more significant increase in inland municipalities (from 4.4% to 76.5%) than in the capitals (from 4.2% to 45.5%),^(^^1^^)^ as well as what was observed in the state of São Paulo, in which the Greater São Paulo Regional Office had a FHS coverage (34.11%), and PHC teams (71.85%) lower than the state average.^(^^6^^)^

As indicated in a study carried out in Espírito Santo, other factors related to the effectiveness of the PHC are pertinent for the professional, such as the fixation on the PHC, improved salary, and specialization in the area. Added to these factors is the availability of hospital beds, with possible encouragement of the use and higher incidence of hospitalizations for ACSC, for reasons such as expediting access to resources or ease of physicians in admitting patients.^(^^13^^)^

The values described here, especially the rates of beds per inhabitants, and the coverage of the FHS and PHC, do not reveal discrepancies between the Regional Offices with an increase in hospitalizations for ACSC spending and those with a reduction. Thus, it is not possible to state, in this study, that the structure of the Health Care Network is the only predictive factor of the variations observed in the hospitalizations for ACSC indicator. Nonetheless, the literature shows the care programs, the structure of the services, and their work process have an impact on the rate of hospitalizations for ACSC.^(^^14^^)^

Analyzing the structure of Primary Health Units in various municipalities of Brazil, the reduction of hospitalizations for ACSC was not identified with the increased coverage of the FHS, raising as possible explanations the overestimated coverage of the FHS, and the care of repressed demand in locations of greater vulnerability.^(^^14^^)^

In a study conducted in Espírito Santo, a reduction in hospitalizations for ACSC associated with the expansion of FHS coverage was observed during the period from 2000 to 2014. Also observed, was the relation between the reduction in hospitalizations for ACSC and the increase in the number of physicians and a greater proportion of users who had completed High School.^(^^13^^)^ The elevation of hospitalizations for ACSC, along with the increased supply of beds, greater coverage of health insurance plans, and greater social inequality, measured by the Gini index, were also noted.^(^^13^^)^ The analysis of hospitalizations for ACSC from 2010 to 2014 in the state of Ceará is consistent with such findings, by associating the increase in hospitalizations for ACSC with low education, high unemployment rates, low income levels, and smaller populations.^(^^3^^)^

In Itaboraí, the reduction of hospitalizations for ACSC was seen with the decline of private hospitals contracted by SUS, without the apparent redistribution of these admissions, speculating the real need for the previous admissions.^(^^10^^)^

Access is not guaranteed only by supply of health services; it is necessary to expand health financing to guarantee the appropriate supply of services,^(^^4^^)^ in addition to investing in the qualification of professionals, and in the establishment of standards and routines in care.^(^^15^^)^

In this context, when observing the increase in FHS coverage and the occurrence of hospitalizations for ACSC, it is important to highlight that it is not enough to expand the coverage of the FHS, without having greater access, capacity of resolution, and quality services.^(^^3^^)^

The reduction of amounts spent on hospitalizations for ACSC in The Regional Offices should be analyzed based on each region's FHS coverage, as presented in this study. Elements related to the organization of health systems and the role played by PHC in the region of interest should be considered.^(^^16^^)^

The expansion of PHC resources observed in the state of São Paulo is a possible element for the reduction in frequency and expenses with hospitalizations for ACSC, although it is necessary to consider other factors, such as social determinants and the organization of health services in each Regional Office.

In conclusion, it is understood the identification of the profile of hospitalizations for ACSC and their financial impact contributed to recognizing the vulnerable users, and points of improvement in the organization and functioning of health services and systems.

As a limitation of this study, we point out the use of SIH data, which makes it impossible to identify people covered or not by the FHS.

## CONCLUSION

The amounts spent on hospitalizations for ambulatory care sensitive conditions presented a reduction. Among the Health Regional Offices, only three presented an increase in expenditures; in the remaining, different proportions of reduction were observed. Significant differences were observed in the amounts spent in the Regional Offices of Greater São Paulo, Barretos, Bauru, Marília, Ribeirão Preto, and São João da Boa Vista.
