# Supplementary material for: Expenses of hospitalization for ambulatory care sensitive conditions in the Health Regional Offices of the State of São Paulo
Source: Einstein (Sao Paulo). 2021 Jul 23;19:eGS5817. doi: 10.31744/einstein_journal/2021GS5817 (PMC8302227; doi:10.31744/einstein_journal/2021GS5817)
Supplement: Translation

# Translation: Gastos com internações por condições sensíveis à Atenção Primária nas Regionais de Saúde do Estado de São Paulo

**Authors:** Bruna Moreno Dias, Ariane Cristina Barboza Zanetti, Antonio Carlos Pereira

**Affiliations:** 1 Ribeirão PretoSP Brasil Escola de Enfermagem de Ribeirão Preto, Universidade de São Paulo, Ribeirão Preto, SP, Brasil.; 2 PiracicabaSP Brasil Faculdade de Odontologia de Piracicaba, Universidade Estadual de Campinas, Piracicaba, SP, Brasil.

**Keywords:** Atenção Primária à Saúde, Hospitalização, Custos hospitalares, Gastos em saúde, Regionalização da saúde

## Abstract

**Objetivo:**

Analisar os gastos com internações por condições sensíveis à Atenção Primária nas Regionais de Saúde do estado de São Paulo.

**Métodos:**

Estudo ecológico, de coorte retrospectiva, com análise de internações hospitalares por condições sensíveis à Atenção Primária ocorridas no estado de São Paulo, no período de 2014 a 2018, computadas pelo Sistema de Informação Hospitalar. Os dados foram extraídos por meio do aplicativo Tabwin e analisados por estatística descritiva.

**Resultados:**

Houve redução de 14,49% do valor gasto com internações por condições sensíveis à Atenção Primária. Foram observadas reduções na frequência das internações (−1,26) e na permanência hospitalar (−0,54) e aumento da ocorrência de óbitos (8,02). As Regionais de Barretos, Taubaté e Araraquara apresentaram elevação de gastos no período, em 37,86%, 15,38% e 3,78%, respectivamente, enquanto todas as demais Regionais apresentaram redução; e as mais expressivas foram em Bauru (−31,90%), São João da Boa Vista (−26,18%), Presidente Prudente (−21,00%) e São Paulo (−19,17%). O valor das internações por condições sensíveis à Atenção Primária apresentou correlação forte e positiva com as variáveis frequência e permanência hospitalar.

**Conclusão:**

Houve diferença de valores gastos com internações por condições sensíveis à Atenção Primária nas regionais, mas não houve diferença na frequência e na duração dessas internações. A expansão de recursos da Atenção Primária à Saúde é um possível elemento para a redução de frequência e dos gastos com internações por condições sensíveis à Atenção Primária; porém é necessário considerar outros fatores, como os determinantes sociais e a organização dos serviços de saúde.

## INTRODUÇÃO

A Atenção Primária à Saúde (APS) é entendida como a principal estratégia para ampliação de acesso e mudança do modelo assistencial do Sistema Único de Saúde (SUS). É também estruturada para ser o eixo norteador desse sistema.^(^^1^^)^

Dentre os atributos da APS, está a capacidade resolutiva, atrelada às medidas de promoção da saúde, prevenção de doenças e tratamento, de modo que a detecção precoce de doenças e seu tratamento adequado não progridam para serviços de outros níveis de atenção, reduzindo o número de internações. Esse é o fundamento que sustenta a proposição do indicador de internações por condições sensíveis à Atenção Primária (ICSAP).^(^^2^^)^

As ICSAP podem ser utilizadas como parte da avaliação da resolubilidade, da qualidade e do acesso à APS, auxiliando na identificação de necessidade, reorientação e proposição de políticas públicas de saúde. De forma complementar, o indicador pode refletir no uso inadequado dos serviços de saúde,^(^^3^^)^ ou ainda evidenciar mudanças no modelo assistencial e no padrão de financiamento em saúde.^(^^4^^)^

O indicador tem sido utilizado em vários países. No Brasil, desde 2008, foi proposta a lista brasileira de ICSAP, que está estruturada em grupos de causas de internações e diagnósticos, com o objetivo de ser um instrumento de avaliação da APS e/ou da utilização da atenção hospitalar, além de desempenho do sistema de saúde nas diferentes esferas de gestão.^(^^5^^)^

A avaliação do sistema de saúde deve ocorrer em paralelo à discussão e à compreensão do financiamento em saúde, que tem sido insuficiente para assegurar um sistema universal, integral e de qualidade. Diante dessa necessidade, compreender o desempenho dos serviços de saúde e a relação com gastos em saúde se mostra como importante possibilidade de otimização do sistema. Ademais, no âmbito das ICSAP, a redução do gasto com internações hospitalares permite o reinvestimento no sistema.^(^^4^^)^

A lista brasileira de condições sensíveis compreende diagnósticos de importante relevância na Atenção à Saúde da população. Assim, ao analisar a realidade de cada região, o monitoramento do perfil e as estratégias de controle e redução de ICSAP possibilitam a adoção de medidas de reestruturação da conformação e o funcionamento dos serviços hospitalares, no enfrentamento de demandas de elevada complexidade assistencial.

Desta forma, a análise de recursos utilizados em ICSAP visa à oferta de subsídios para ações de planejamento, gestão e avaliação de serviços, programas e políticas de saúde – em especial para as situações de maior vulnerabilidade e iniquidade.

## OBJETIVO

Analisar os gastos com internações por condições sensíveis à Atenção Primária nas Regionais de Saúde do Estado de São Paulo.

## MÉTODOS

Estudo ecológico, de coorte retrospectiva, tendo como desfecho a ocorrência de internação hospitalar de usuários com diagnóstico principal constante na lista brasileira de condições sensíveis à APS nas Regionais de Saúde do Estado de São Paulo.

Foram consideradas elegíveis todas as internações ocorridas no estado de São Paulo no período de 2014 a 2018, computadas pelo Sistema de Informações Hospitalares (SIH) do SUS, com dados estruturados nas 17 Regionais de Saúde do estado: Grande São Paulo; Araçatuba; Araraquara; Baixada Santista; Barretos; Bauru; Campinas; Franca; Marília; Piracicaba; Presidente Prudente; Registro; Ribeirão Preto; São João da Boa Vista; São José do Rio Preto; Sorocaba e Taubaté.

Foi considerado o período de cinco anos, tendo o ano de 2018 como o mais recente de dados consolidados disponíveis para consulta pública, na ocasião da busca. O ano de 2014 foi considerado como referência para ajuste de valores, segundo a inflação do período.

Utilizaram-se dados de fonte secundária, originários dos microdados do SIH do Departamento de Informática do SUS (DATASUS), por meio de consulta pública, sob forma de arquivos reduzidos, e o aplicativo Tabwin versão 4.1.5, desenvolvido e disponibilizado pelo DATASUS.

A identificação de internações por condições sensíveis se baseou na relação de diagnósticos segundo a Classificação Internacional de Doenças e Problemas Relacionados à Saúde (CID-10), constantes na lista brasileira de ICSAP, publicada pela portaria 221 do Ministério da Saúde, de 17 de abril de 2008.^(^^5^^)^

Os registros obtidos foram exportados para o software Microsoft Excel, compondo banco de dados em planilha eletrônica. Para análise estatística, foi utilizado o software IBM SPSS Statistics, versão 19. Foi utilizada a correlação linear, para análise da relação entre variáveis numéricas; e, para comparação entre os anos 2014 e 2018, foi utilizado o teste pareado de Wilcoxon, com nível de significância de 5%.

## RESULTADOS

No período em estudo, observou-se a elevação das internações gerais e aumento da população do estado, conforme ocorreu a redução das ICSAP (Tabela 1).

**Tabela 1:** Proporção e taxa de internações por condições sensíveis à Atenção Primária no Estado de São Paulo

Ano	ICSAP	Internações gerais	Proporção ICSAP (%)	População	Taxa ICSAP (por mil)
2014	377.988	2.496.141	15,14	43.937.755	8,60
2015	377.752	2.493.368	15,15	44.356.304	8,52
2016	372.974	2.489.614	14,98	44.760.305	8,33
2017	373.069	2.498.888	14,93	45.149.603	8,26
2018	373.241	2.538.337	14,70	45.538.936	8,20

ICSAP: internações por condições sensíveis à Atenção Primária.

Os valores gastos com ICSAP comprometeram 12,72% do valor das internações gerais. Conforme pode ser observado na figura 1, as Regionais com menores proporções de gastos em relação às internações gerais foram Barretos, Bauru e São João da Boa Vista; as de maiores proporções foram Araraquara, Araçatuba e Presidente Prudente.

**Figura 1:** Proporção de gastos com internações por condições sensíveis à Atenção Primária em relação aos gastos com internações gerais em 2018

Em termos absolutos, a frequência de ICSAP reduziu em 11 das 17 Regionais, sendo as com maior redução Bauru, São João da Boa Vista e Piracicaba. A elevação de frequência se deu, principalmente, nas Regionais Taubaté e Araraquara, conforme apresentado na tabela 2. Ao comparar os anos de 2014 e 2018, não foi observada diferença significativa na frequência de ICSAP (p=0,435).

**Tabela 2:** Frequência, permanência, óbitos e valores gastos com internações por condições sensíveis à Atenção Primária por Regional de Saúde no Estado de São Paulo

Regionais de Saúde	Frequência				Valor (R$)				Permanência				Óbito			
	2014	2018	Variação(%)	Valor de p*	2014†	2018	Variação(%)	Valor de p*	2014	2018	Variação(%)	Valor de p*	2014	2018	Variação(%)	Valor de p*
Grande São Paulo	151.404	154.241	1,87	0,717	225.575.520,73	182.330.045,28	−19,17	0,005‡	1.107.765	1.050.257	−5,19	0,398	9.167	9.991	8,99	0,396
Araçatuba	9.179	8.505	−7,34	0,794	8.696.477,00	8.522.201,50	−2,00	0,198	39.109	41.163	5,25	0,421	584	625	7,02	0,849
Araraquara	10.418	11.489	10,28	0,121	14.189.798,98	14.726.521,44	3,78	0,277	76.060	69.921	−8,07	0,184	746	778	4,29	0,192
Baixada Santista	10.470	9.914	−5,31	0,494	12.191.048,68	12.117.636,75	−0,60	0,968	73.034	75.188	2,95	0,778	930	1.109	19,25	0,067
Barretos	5.425	5.197	−4,20	0,184	3.688.118,69	5.084.465,88	37,86	0,024‡	21.402	26.623	24,39	0,103	272	362	33,09	0,199
Bauru	23.963	19.894	−16,98	0,014‡	30.225.019,28	20.583.674,20	−31,90	0,005‡	132.591	120.284	−9,28	0,147	1.488	1.306	−12,23	0,133
Campinas	29.171	30.844	5,74	0,117	39.535.158,53	34.331.818,73	−13,16	0,126	174.020	190.628	9,54	0,036‡	1.806	1.855	2,71	0,672
Franca	7.024	6.965	−0,84	0,717	10.141.339,43	8.417.055,31	−17,00	0,243	30.425	30.921	1,63	0,872	477	387	−18,87	0,033‡
Marília	16.616	15.180	−8,64	0,334	17.601.805,86	15.780.963,18	−10,34	0,030‡	83.783	88.190	5,26	0,376	1.026	1.001	−2,44	0,460
Piracicaba	10.146	9.010	−11,20	0,067	16.382.931,56	13.300.291,47	−18,82	0,126	56.661	52.696	−7,00	0,376	720	731	1,53	0,536
Presidente Prudente	13.314	13.512	1,49	0,841	14.014.865,71	11.071.972,62	−21,00	0,099	60.237	61.590	2,25	0,546	733	712	−2,86	0,950
Registro	1.911	1.810	−5,29	0,931	1.493.468,36	1.376.145,93	−7,86	0,687	10.682	11.551	8,14	0,171	273	354	29,67	0,043‡
Ribeirão Preto	15.184	15.744	3,69	0,421	26.336.769,31	22.298.947,30	−15,33	0,005‡	88.465	94.897	7,27	0,243	834	842	0,96	0,652
São João da Boa Vista	10.557	9.138	−13,44	0,023‡	8.568.923,81	6.325.339,92	−26,18	<0,001‡	47.913	45.956	−4,08	0,520	850	719	−15,41	0,170
São José do Rio Preto	26.039	23.477	−9,84	0,212	37.273.150,84	34.250.673,03	−8,11	0,968	109.751	115.225	4,99	0,629	924	1.440	55,84	0,024‡
Sorocaba	18.265	17.112	−6,31	0,573	20.268.370,15	17.507.823,74	−13,62	0,070	85.730	93.699	9,30	0,398	1.657	1.770	6,82	0,338
Taubaté	18.902	21.209	12,21	0,243	25.885.826,35	29.866.293,97	15,38	0,968	146.653	162.927	11,10	0,084	1.434	1.858	29,57	0,016‡
Total	377.988	373.241	−1,26	0,435	512.068.592,46	437.891.870,25	−14,49	0,010‡	2.344.281	2.331.716	−0,54	0,266	23.921	25.840	8,02	0,107

* Teste pareado de Wilcoxon, considerada a significância estatística para valores de p<0,05

† valor de 2014 ajustado pela inflação no período

‡ valores de p<0,05.

Para os valores gastos, observou-se diferença significativa entre os anos em estudo (p=0,010), com redução na ordem de −14,49%, quando comparado o valor gasto em 2018 com o valor gasto em 2014, ajustado para inflação. As Regionais de Barretos, Taubaté e Araraquara foram as únicas com elevação de gastos no período, em 37,86%, 15,38% e 3,78%, respectivamente. Já as reduções mais expressivas ocorreram em Bauru (−31,90%), São João da Boa Vista (−26,18%), Presidente Prudente (−21,00%) e São Paulo (−19,17%). Observou-se diferença significativa para os valores gastos nas regiões da Grande São Paulo (valor de p=0,005), Barretos (valor de p=0,024), Bauru (valor de p=0,005), Marília (valor de p=0,030), Ribeirão Preto (valor de p=0,005) e São João da Boa Vista (valor de p<0,001).

Embora tenha ocorrido ligeira redução na permanência hospitalar (−0,54%), algumas Regionais apresentaram elevação superior a 10 pontos percentuais, como Barretos (24,39%) e Taubaté (11,10%). As Regionais com maiores reduções de permanência foram Bauru (−9,28%), Araraquara (−8,07%) e Piracicaba (−7,00%).

Observou-se elevação da ocorrência de óbitos no estado, sendo as Regionais com elevações mais expressivas São José do Rio Preto (55,84%), Barretos (33,09%), Registro (29,67%) e Taubaté (29,57%). Por sua vez, as reduções se deram em Franca (−18,87%) e São João da Boa Vista (−15,41%).

Não foram observadas diferenças significativas entre os anos de 2014 e 2018 no tempo de permanência hospitalar (valor de p=0,266) e nem na ocorrência de óbitos (valor de p=0,107).

As Regionais de Bauru e São João da Boa Vista apresentaram comportamento uniforme da redução de frequência, valor, permanência hospitalar e óbitos. Taubaté apresentou elevação em todas as variáveis e Barretos, embora tenha apresentado redução na frequência de internações, teve elevação nas demais variáveis, sendo a Regional com elevações mais expressivas do valor gasto e do tempo de permanência hospitalar.

Ainda, tomando como referência o ano de 2018, foi identificada correlação forte e positiva entre a frequência e o valor das ICSAP (r=0,997), entre a permanência hospitalar e o valor das ICSAP (r=0,996) e entre a frequência de internações e a ocorrência de óbitos por condições sensíveis à Atenção Primária (r=0,994).

## DISCUSSÃO

No período em estudo, observou-se redução da proporção (de 15,14 para 14,70) e da taxa de ICSAP (de 8,60 para 8,20) simultaneamente à expansão de capacidade instalada da APS, evidenciada pelo aumento do número de equipes de Estratégia Saúde da Família (ESF), de 3.380, em 2010, para 5.257, em 2018, além de maiores coberturas, no mesmo período, de equipes de ESF e equipes de Atenção Primária – de 28,29% para 41,23% e de 82,55 para 85,96%, respectivamente.^(^^6^^)^

As taxas observadas se mostram similares a cidades com altas coberturas de Atenção Primária, como Florianópolis e Curitiba, com valores próximos a 10%, enquanto o Distrito Federal apresentou valor próximo a 13%.^(^^7^^)^

A proporção de ICSAP em relação às internações gerais também se mostra inferior a de outros estudos, como observado em Goiás, local em que 30% das internações se deram por condições sensíveis à Atenção Primária,^(^^8^^)^ 25,3% em municípios do Mato Grosso do Sul^(^^9^^)^ e 32,4% no município de Itaboraí (RJ).^(^^10^^)^

Em 2018, as ICSAP corresponderam por 12,72% dos gastos totais em internações. Esse comportamento também foi observado em São Leopoldo (RS), que, em 2012, destinou 15,80% dos gastos com internações em ICSAP.^(^^11^^)^

Merece destaque a elevação de valores das Regionais de Barretos, Taubaté e Araraquara. Na Regional de Barretos, observa-se elevação do valor gasto, da permanência e dos óbitos por condições sensíveis à Atenção Primária, embora tenha ocorrido a redução na frequência de internações. A Regional conta com 2,05 leitos para cada mil habitantes, ou seja, uma maior proporção dentre as Regionais, além de cobertura de 58,81% de equipes de ESF e cobertura de 107,24% de APS.^(^^6^^)^ Apesar da elevação do valor gasto em ICSAP, a Regional comprometeu, no ano de 2018, 7,89% do valor gasto com internações gerais, que é a menor proporção observada entre as Regionais.

Na Regional de Taubaté, houve elevação de valores gastos, acompanhada por elevação de frequência, permanência e óbitos por condições sensíveis à Atenção Primária. A regional possui 1,29 leito por mil habitantes, sendo 48,37% de cobertura de ESF e 96,38% de cobertura de APS;^(^^6^^)^ comprometendo 16,13% de valores gastos em ICSAP.

De forma similar, a Regional de Araraquara apresentou redução na permanência de ICSAP, embora tenha elevado frequência, valor e óbitos. A Regional conta com 1,38 leito por mil habitantes, tem cobertura de ESF de 45,84% e cobertura de Atenção Primária de 99,65%.^(^^6^^)^ É a Regional com maior proporção de gastos, quando comparados os valores de internações gerais, com 21,51%.

Dentre as Regionais em que houve redução dos valores gastos, cabe destaque para Bauru e São João da Boa Vista. Em ambas as Regionais, a redução de valores gastos acompanha a diminuição da frequência, da permanência e dos óbitos por ICSAP.

Em São João da Boa Vista, há 1,48 leito por mil habitantes, e a cobertura de ESF é de 46,4% e a cobertura de APS é de 92,65%.^(^^6^^)^ A proporção do valor de ICSAP entre as internações gerais foi de 10,33%. Já em Bauru, há 1,88 leito por mil habitantes, 43,35% de cobertura de ESF e 102,45% de cobertura de APS.^(^^6^^)^ A proporção do valor de ICSAP entre as internações gerais foi de 9,87%.

Em estudo realizado previamente, nas Regionais do estado de São Paulo, no período de 2000 a 2007, houve redução de frequência de ICSAP em quase todas as Regionais, exceto Grande São Paulo, Araçatuba e Ribeirão Preto.^(^^12^^)^ Neste estudo, São Paulo e Ribeirão Preto permaneceram em movimento de elevação de frequência de ICSAP, enquanto Araçatuba apresentou redução. Para o período de 2000 a 2007, foram relatadas as maiores reduções nas Regionais de Barretos, Araraquara e Taubaté,^(^^12^^)^ que, neste estudo, com exceção de Barretos, sofreram elevação da frequência.

Em termos de estrutura, nota-se, em todas as Regionais, a ampliação de recursos da APS, com ampliação de cobertura de ESF de 28,29%, em 2010, para 41,23%, em 2018, e importante diferenças entre as Regionais, conforme é possível observar nos números mínimos e máximos para o ano de 2018, nas Regionais em estudo, para números de ESF (72 a 2.062), cobertura de ESF (30,46% a 108,29%), número de médicos na APS (130 a 4.995) e cobertura de equipes de APS (71,85% a 140,70%).^(^^6^^)^

Esse comportamento acompanha a expansão de equipes de APS no país nos últimos 20 anos, em que a cobertura de ESF passou de 4,4% a 70%, sendo a elevação mais expressiva nos municípios do interior (de 4,4% para 76,5%) que nas capitais (de 4,2% para 45,5%),^(^^1^^)^ assim como observado no estado de São Paulo, em que a Regional Grande São Paulo apresenta cobertura de ESF (34,11%) e de equipes de APS (71,85%) inferior à média do estado.^(^^6^^)^

Outros fatores relacionados à efetividade da APS, apontados em estudo realizado no Espírito Santo, são pertinentes aos profissional, como a fixação na APS, a melhoria de salário e a especialização na área. Soma-se a esses fatores a disponibilidade de leitos hospitalares, com possível indução à utilização e maior incidência de ICSAP, por razões como agilidade de acesso aos recursos ou facilidade dos médicos em internar os usuários.^(^^13^^)^

Os valores aqui descritos, em especial as taxas de leitos por habitantes, e as coberturas de ESF e APS, não revelam discrepâncias entre as Regionais que apresentaram elevação do gasto em ICSAP e aquelas em que houve redução. Assim, não se pode afirmar, neste estudo, que a estrutura da Rede de Atenção à Saúde seja o único fator preditivo das variações observadas no indicador de ICSAP. Entretanto, observa-se na literatura que os programas assistenciais, a estrutura dos serviços e seu processo de trabalho impactam nas taxas de ICSAP.^(^^14^^)^

Em análise da estrutura das Unidades Básicas de Saúde de diversos municípios do Brasil, não foi identificada a redução das ICSAP com a elevação da cobertura de ESF, levantando como possíveis explicações a cobertura superestimada da ESF, e o atendimento de demanda reprimida, em localidades de maior vulnerabilidade.^(^^14^^)^

Já em estudo realizado no Espírito Santo, foi observada a redução de ICSAP associada à expansão de cobertura de ESF, no período de 2000 a 2014. Além disso, também foi observada a relação entre a redução de ICSAP e o aumento da proporção de médicos e maior proporção de usuários com ensino médio.^(^^13^^)^ A elevação de ICSAP, com aumento da oferta de leitos, maior cobertura de planos de saúde suplementar e maior desigualdade social, mensurada pelo índice Gini, também foi notada.^(^^13^^)^ A análise de ICSAP no período de 2010 a 2014 no estado do Ceará coaduna tais achados, ao associar a elevação de ICSAP com a baixa escolaridade, as altas taxas de desemprego, os baixos níveis de renda e os menores aportes populacionais.^(^^3^^)^

Em Itaboraí foi observada a redução de ICSAP com o declínio de hospitais privados contratados pelo SUS, sem a aparente redistribuições dessas internações, especulando a real necessidade das internações realizadas anteriormente.^(^^10^^)^

O acesso não é garantido apenas pela oferta de serviços de saúde, sendo necessário ampliar o financiamento em saúde, de forma a garantir a oferta adequada de serviços,^(^^4^^)^ além de investir na qualificação dos profissionais e no estabelecimento de padrões e de rotinas nos atendimentos.^(^^15^^)^

Nesse âmbito, ao observar a elevação de cobertura de ESF e da ocorrência de ICSAP, destaca-se que não basta ampliar a cobertura de ESF, sem que existam maior acesso, resolubilidade e qualidade dos serviços.^(^^3^^)^

A redução de valores gastos nas ICSAP nas regiões de saúde deve ser analisada com base na cobertura de ESF de cada região, como apresentado neste estudo. Também devem ser considerados elementos relacionados à organização dos sistemas de saúde e ao papel desempenhado pela APS na região de interesse.^(^^16^^)^

A expansão de recursos da APS observada no estado de São Paulo é um possível elemento para a redução de frequência e gastos com ICSAP, embora seja necessário considerar outros fatores, como os determinantes sociais e a organização dos serviços de saúde em cada Regional.

Por fim, entende-se que a identificação do perfil de ICSAP e seu impacto financeiro contribuem para o reconhecimento de usuários em vulnerabilidade e de pontos de melhoria na organização e no funcionamento de serviços e sistemas de saúde.

Como limitação deste estudo, aponta-se a utilização de dados do SIH, o que impossibilita a identificação de pessoas cobertas ou não pela ESF.

## CONCLUSÃO

Os valores gastos com internações por condições sensíveis à Atenção Primária apresentaram redução. Dentre as Regionais de Saúde, apenas três apresentaram elevação dos gastos; nas demais, foram observadas diferentes proporções de redução. Foram observadas diferenças significativas nos valores gastos nas regiões de Grande São Paulo, Barretos, Bauru, Marília, Ribeirão Preto e São João da Boa Vista.
